# Dr. Edward Pharr

**Published:** 1876-08

**Authors:** 


DR. EDWARD PHARR.—We are pained to have to record the death of this good man. He died some months ago, in Troup county, Georgia, of disease of the kidneys, supposed to be the result of exposure and fatigue incident to an extensive practice in the country. Dr. Pharr had lived in Troup county for near a quarter of a century, and was surrounded by a host of friends who mourn his loss. *Requiescat in pace.*

